# Childhood Maltreatment and Adolescent Risky Behavior: Mediating the Effect of Parent–Adolescent Conflict and Violent Tendencies

**DOI:** 10.3390/bs14111058

**Published:** 2024-11-06

**Authors:** Serap Özdemir Bişkin

**Affiliations:** Department of Psychological Counselling and Guidance, Faculty of Education, Burdur Mehmet Akif Ersoy University, 15030 Burdur, Turkey; sozdemir@mehmetakif.edu.tr

**Keywords:** childhood maltreatment, adolescent risky behavior, parent–adolescent conflict, violent tendency

## Abstract

Childhood maltreatment experiences are an important public health issue associated with a variety of short- and long-term social and psychological outcomes. Despite the negative impact of these experiences, little research has examined the mechanisms underlying the relationship between childhood maltreatment and adolescent risky behavior, which is a critical step in developing intervention services to prevent such behavior during adolescence. The present study aimed to examine the mediating role of parent–adolescent conflict and violent tendency in the relationships between childhood maltreatment and adolescent risky behavior in adolescence. Participants were 303 high school students, 60.1% female (*n* = 182) and 39.9% (*n* = 121) male, from four different high schools in Türkiye. It was found that childhood maltreatment was significantly and positively associated with adolescent risky behavior. In addition, that parent–adolescent conflict and violent tendencies mediated the relationship between childhood maltreatment and adolescent risky behavior. And also parent–adolescent conflict mediated the relationship between childhood maltreatment and violent tendencies and adolescent risky behavior. The results of this study suggest that parent–adolescent conflict and violent tendencies are important in increasing the impact of childhood maltreatment on adolescent risky behavior. In order to reduce the impact of maltreated childhood experiences on risky behavior, interventions can be designed to increase positive adolescent–parent relationships and reduce violent tendencies.

## 1. Childhood Maltreatment and Adolescent Risky Behavior

Childhood maltreatment represents a significant global public health issue, encompassing various forms of abuse and neglect—including physical, emotional, and sexual abuse, as well as exploitation—that pose risks to a child’s health, development, and dignity within contexts of trust or authority [1]. Physical abuse involves the intentional use of force, resulting in harm through actions like hitting, shaking, or burning. Emotional abuse, on the other hand, includes behaviors that diminish a child’s self-worth or emotional well-being, such as verbal abuse, constant criticism, rejection, or isolation. Sexual abuse occurs when a child is coerced or enticed into sexual activities, which may include physical contact or exposure to inappropriate material. Neglect, one of the most prevalent forms of maltreatment, refers to the failure of caregivers to meet a child’s basic needs, including food, shelter, and healthcare [1]. Research consistently has shown that childhood maltreatment has profound developmental consequences, leading to adverse psychological and social outcomes such as low self-esteem, emotional dysregulation, and difficulties in interpersonal relationships [2,3,4,5]. Furthermore, maltreated children are at increased risk for mental health disorders, including depression, anxiety, and post-traumatic stress disorder [6,7,8], and are more likely to engage in risky behaviors during adolescence [8,9,10,11,12,13].

Risky behaviors in adolescence have been defined as “any consciously or unconsciously controlled behavior with a perceived uncertainty about its outcome, and/or about its possible benefits or costs for the physical, economic, or psychosocial well-being of oneself or others” [14]. Such behaviors, including substance use, early sexual activity, antisocial behavior, suicide, dropping out of school, and unhealthy eating pose significant threats to adolescent health, safety, and well-being [15] Different forms of maltreatment are associated with varying degrees of risk for adolescents to engage in risky behaviors. Physical abuse is often linked to externalizing behaviors like aggression and delinquency, with studies indicating that adolescents who experience physical maltreatment are more likely to exhibit such behaviors [16,17]. Emotional abuse, on the other hand, has been correlated with internalizing issues, including anxiety and depression, which may lead to substance use, suicidal ideation, and unsafe sexual practices [8,18,19,20]. Adolescents who have endured sexual abuse are at heightened risk for self-harm, substance use, and risky sexual behaviors [18,21,22]. Neglect, another form of maltreatment, can result in developmental delays and socialization challenges, increasing the likelihood of substance use and other risky behaviors due to impaired social skills and emotional regulation [23,24]. The long-term impacts of childhood maltreatment on family dynamics and behavioral patterns often persist into adulthood.

General strain theory (GST) [25,26] offers a valuable framework for understanding the relationship between childhood maltreatment and risky behaviors. Agnew [26] argues that victimization, especially child abuse and neglect, is one of the most consequential types of strain. It posits that strain leads to negative emotions such as anger and frustration, which can, in turn, lead to deviant behaviors as individuals seek to cope with these emotions. The theory suggests that not all individuals respond to strain in the same way; rather, their responses are affected by their coping mechanisms. It is essential to identify different cultural factors that may affect this relationship to develop effective intervention programs aimed at preventing risky behaviors. Therefore, the present study seeks to examine the mediating roles of parent–adolescent conflict and violent tendencies in the relationship between childhood maltreatment and risky behavior in Turkish adolescents.

## 2. Parent–Adolescent Conflict

Parent–adolescent conflict, characterized by disagreements, arguments, and tensions between adolescents and their parents, is a normal part of adolescent development [27]. However, when these conflicts become chronic and intense, they are often associated with psychological distress and an increase in risky behaviors among adolescents [4,28,29,30]. For example, Lam et al. [31] found that adolescents who experienced higher levels of conflict with their parents reported increased engagement in risky behaviors compared to their siblings. Similarly, Liu et al. [32] identified that both increased parent–adolescent conflict and lower levels of perceived parental support predicted greater involvement in risky behaviors. Additionally, Chaplin et al. [28] suggested that low levels of warm and supportive parenting during conflicts may heighten adolescent anger, leading to risky behaviors such as alcohol use. Ultimately, prolonged parent–adolescent conflict, particularly in the absence of parental warmth and support, plays a critical role in increasing adolescents’ propensity to engage in risky behaviors.

Children who have experienced maltreatment—whether physical, emotional, or sexual—are more prone to frequent and intense conflicts with their parents [4,33]. Adolescents with a history of early maltreatment are likely to exhibit high emotional reactions toward their parents, such as anger and frustration, which can negatively affect the parent–adolescent bond and escalate conflicts during adolescence [33,34]. Moreover, early childhood maltreatment experiences can undermine trust and communication within the family, weakening the parent–child relationship, parental monitoring and further diminishing parental support [18,35,36]. Longitudinal studies have shown that maternal childhood neglect and abuse are associated with lower levels of emotional closeness with family members in adulthood, which in turn increase risky behaviors such as substance use or self-harm [5,37]. For example, Yoon et al. [22] found that emotional abuse and neglect were associated with poor mother–child relationship quality, which predicted adolescent substance use.

According to Hirschi’s [38] social control theory, deviant behavior can be controlled through strong social bonds. Therefore, when adolescents have strong bonds with their parents, the risk of engaging in deviant behavior decreases accordingly. Turkish culture also places a strong emphasis on family unity, so parent–adolescent conflict can undermine traditional social controls, which can be a risk factor for risky behaviors. In this context, this study aims to explore how early maltreatment experiences disrupt parent–child relationships and increase the likelihood of risky behaviors during adolescence, while also exploring how strong social bonds with parents can serve as protective factors against deviant behaviors.

## 3. Violent Tendencies

Violent tendencies in adolescence refer to an adolescent’s propensity to engage in aggressive or violent acts. They often manifest as expressions of internalized anger and can be viewed as a coping or problem-solving strategy developed through social learning [24,25,26,39]. Bandura’s [40] social learning theory suggests that such tendencies are often acquired through observation and imitation of aggression in one’s environment. In an environment where violence is normalized, such as abusive families, adolescents may increasingly turn to violence to cope with challenges with parents or peers, normalizing aggressive responses over time without healthier strategies to manage emotions such as anger. Therefore, for those who grow up in traumatic or abusive environments, the presence of violent role models and the absence of supportive adults can strengthen violent tendencies [4,41,42]. Thus, research has shown that chronic stress and trauma from childhood maltreatment can increase violent tendencies and behavior [8,24,34,41,43].

Violent tendencies often lead to negative outcomes such as reduced social support [41], decreased school engagement [44], and increased loneliness, which in turn can lead to further violence [41,45]. Dodge et al. [46] suggest that maltreated children often develop maladaptive patterns of processing social cues that may promote aggressive behavior. This aggression, shaped by ineffective coping strategies, may escalate into broader risky behaviors such as substance abuse or antisocial acts [46,47]. Thus, adolescents exposed to maltreatment and parental conflict may adopt violence as a problem-solving approach, increasing their likelihood of engaging in various risky behaviors. An effective intervention could help equip these adolescents with healthier coping strategies and reduce the risk of maladaptive behaviors.

## 4. The Present Study

The present study aims to investigate the relationship between childhood maltreatment and adolescent risky behavior by examining the mediating roles of parent–adolescent conflict and violent tendencies. GST posits that individuals exposed to strain are more likely to engage in deviant behaviors when social controls are weakened or when they learn maladaptive coping mechanisms, such as violence, through social learning [26]. Within the scope of this study, it is expected that childhood maltreatment constitutes strain and this strain transforms into risky behaviors through parent–adolescent conflict and violent tendencies. Parent–adolescent conflicts may weaken social controls within the family, while violent tendencies, which are learned responses, may cause adolescents to develop aggressive responses to conflicts. These two variables may play a role in the transformation of strain into risky behaviors, as predicted by GST. Previous studies have addressed the relationships between strain and deviant behavior within the framework of GST, but there is still a need to identify different factors that may mediate this relationship to better understand it.

The results of the current study will provide in-depth insights into understanding the impact of family dynamics on adolescent risky behaviors, especially in Turkey, and will offer significant contributions to previous research in this area. Moreover, it will provide practical insights on how to address parent–adolescent dynamics and violent tendencies in the development of intervention strategies. The results may contribute to the development of policies and interventions aimed at strengthening family communication and support mechanisms. In line with these objectives, the following hypotheses were proposed. H1: childhood maltreatment will be positively associated with adolescent risky behavior. H2: parent–adolescent conflict will mediate the relationship between childhood maltreatment and both violent tendencies and adolescent risky behavior. H3: violent tendencies will mediate the relationship between childhood maltreatment and adolescent risky behavior

## 5. Method

### 5.1. Participants

This study included 303 high school students enrolled in four different high schools in Türkiye. The sample was 60.1% female (*n* = 182) and 39.9% male (*n* = 121) and the student distribution across different grade levels was as follows: 19.5% first grade (59 students), 19.8% second grade (60 students), 44.9% third grade (136 students), and 15.8% fourth grade (48 students). The mean age of the participants was 16.5 years (SD = 0.97), ranging from 15 to 18 years old. A priori power analysis was conducted using G* Power version 3.1.9.7. to determine the required sample size for a medium effect size (f_2_ = 0.15) with an alpha level of 0.05 and a desired power of 0.95. The analysis indicated that a minimum of 119 participants would be required to detect a statistically significant effect with adequate power. The data collection process was conducted during class periods at times and dates deemed appropriate by the teachers. Researchers entered the classrooms to administer the surveys, ensuring that the process was carried out smoothly and with minimal disruption to the students’ regular activities. This method allowed for a comprehensive and direct engagement with the participants, ensuring the reliability and validity of the data collected. To ensure that data collection adhered to ethical principles, the parental informed consent form was obtained from parents. This study adhered to the ethical principles of the 1964 Declaration of Helsinki. Participants were not offered any incentives for their involvement in the study.

### 5.2. Measures

Childhood Maltreatment Scale (CMS): The CMS was developed by Bernstein et al. [48] to measure experiences of childhood maltreatment. The scale was translated into Turkish by Aslan and Alparslan [49]. This scale consists of a total of 40 items and uses a 5-point Likert scale (1 = never, 5 = very often). High scores indicate more frequent experiences of neglect and abuse in childhood and adolescence. The Cronbach’s alpha coefficient was reported to be 0.94 for physical neglect and abuse, 0.95 for emotional neglect and abuse, 0.94 for sexual abuse, and 0.96 for the total scale [49]. The internal reliability estimate for this sample was 0.91.

Risky Behavior Scale (RBS): The RBS was developed by Gençtanırım-Kuru [50] to measure risky behavior among Turkish adolescents. This scale consists of a total of 36 items and uses a 5-point Likert scale (1 = strongly disagree, 5 = strongly agree). High scores on the RBS indicate more frequent engagement in risky behaviors during adolescence. The scale has six subscales: alcohol use, smoking, antisocial behavior, dropping out of school, suicidal tendencies, and eating habits. Conbach’s alpha coefficient was 0.91 [50] The estimate of internal reliability for this sample was 0.92.

Violence Tendency Scale (VTS): The VTS was developed by Haskan and Yıldırım [51] to determine the violent tendencies of secondary school students. This scale consists of a total of 20 items and each item is evaluated with a 3-point rating scale (1 = never, 2 = sometimes, 3 = always). High scores obtained from the scale indicate that the individual has a high tendency for violence. The reliability of the scale was measured by Cronbach’s alpha coefficient and was determined as 0.87 [51]. The estimate of the internal reliability for this sample was 0.87.

The Adolescent–Parent Conflict Scale (APCS): The APCS was developed by Eryılmaz and Mammadov [52] to assess the level of conflict between adolescents and their parents. This scale, which is based on adolescent self-report, consists of a total of 12 items and uses a 4-point Likert-type rating system (1 = not at all, 4 = very much). High scores on the scale indicate a high level of conflict between the adolescent and parents. The Cronbach’s alpha coefficient was found to be 0.88, which indicates that the scale has a high internal consistency [52]. The internal reliability estimate for this sample was 0.89.

Demographic Information Form: A demographic information form was used to collect demographic information, including participants’ gender, grade level, and age.

### 5.3. Data Analyses

Prior to testing the proposed mediation models, preliminary analyses were conducted to evaluate descriptive statistics, assess normality, and examine correlation coefficients between the study variables. Normality was assessed based on kurtosis and skewness values, adhering to established cutoffs [53]. Next, Pearson’s product-moment correlation analysis was conducted to examine the relationships among the study variables. Next, parallel and serial mediation analyses were carried out to test the mediating roles of parent–adolescent conflict and violent tendencies in the relationship between childhood maltreatment and adolescent risky behavior. To test the mediation model, model 6 was tested for serial mediation using PROCESS macro v4.2 [54] for SPSS. Regression-based mediation methods, particularly those using bootstrapping techniques, have been shown to provide reliable and valid estimates of indirect effects, especially with small to medium sample sizes [55]. Model results were interpreted based on standardized path estimates (*β*) and squared multiple correlations (R^2^). Additionally, the bootstrap method using 5000 replicate samples was used to calculate 95% confidence intervals (CI) for indirect effects (Hayes [54]). All statistical analyses were performed using SPSS version 26.

## 6. Results

Descriptive statistics demonstrated that skewness and kurtosis scores ranged between −0.41 and 2.51, which suggests that all variables of the study were relatively normally distributed (skewness < |1.5|, and kurtosis < |3|), as shown in Table 1. Then, the correlation analysis was conducted to explore the associations between the study variables. The findings showed that childhood maltreatment had significant and positive correlations with adolescent risky behavior. Adolescent childhood maltreatment had also a significant correlation with parent–adolescent conflict and violent tendencies. Additionally, adolescent–parent conflict and violent tendencies had a positive correlation with adolescent risky behavior, as seen in Table 1.

Serial mediation analyses were conducted to determine whether parent–adolescent conflict and violent tendency mediated the association between childhood maltreatment experiences and adolescent risky behavior and whether parent–adolescent conflict mediated the association between childhood maltreatment experiences and violent tendencies in adolescents. Firstly, it was found that childhood maltreatment significantly predicted adolescent risky behavior (*β* = 0.20, *p* < 0.001). Also, childhood maltreatment had a significant and positive effect on parent–adolescent conflict (*β* = 0.24, *p* < 0.001) and violent tendencies (*β* = 0.12, *p* < 0.001), accounting for 7% of the variance in parent–adolescent conflict, as shown in Table 2.

**Figure 1:**
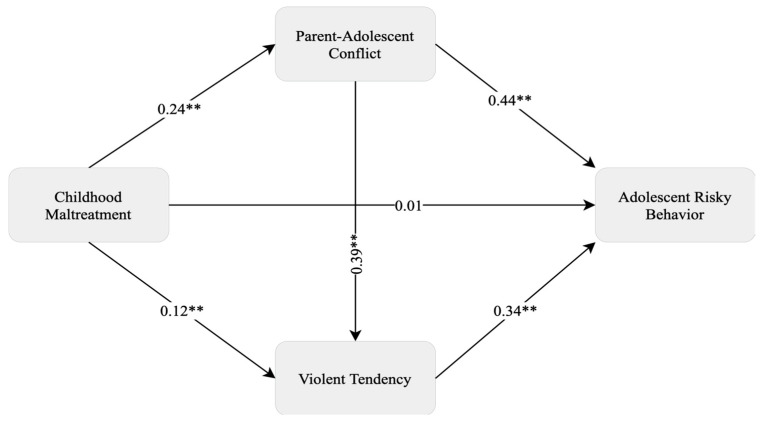
Unstandardized mediating effects of parent–adolescent conflict and violent tendency. ** *p* < 0.01.

Additionally, as seen in Table 2, childhood maltreatment and parent–adolescent conflict together explained 17% of the variance in violent tendencies and childhood maltreatment predicted violent tendencies through parent–adolescent conflict in adolescents. Finally, it was found that childhood maltreatment did not have a significant direct effect on adolescent risky behavior (*β* = 0.01, *p* = 0.76). However, both parent–adolescent conflict (β = 0.44, *p* < 0.001) and violent tendencies (*β* = 0.34, *p* < 0.001) significantly predicted adolescent risky behavior. This model explains 40% of the variance in adolescent risky behavior. As seen in Figure 1 and Table 3, these results suggest that parent–adolescent conflict and violent tendencies play a mediating role between childhood maltreatment experiences and adolescent risky behavior.

Table 3 shows the unstandardized total, direct, and indirect effects with 95% bias-adjusted confidence intervals predicting adolescent risky behavior scores. The analysis shows that childhood maltreatment has a significant indirect effect on adolescent risky behavior through parent–adolescent conflict and violent tendencies (*β* = 0.18). The findings highlight that childhood abuse is indirectly associated with adolescent risky behavior through the role of parent–adolescent conflict (*β* = 0.10, *p* < 0.01) and violent tendencies (*β* = 0.04, *p* < 0.01). Lastly, the serial mediation model demonstrates that childhood maltreatment predicts adolescent–parent conflict, which in turn predicts violent tendencies, and ultimately leads to adolescent risky behavior. It suggests that childhood abuse strengthens adolescents’ risky behavior by first increasing parent–adolescent conflict and then developing violent tendencies.

## 7. Discussion

The present study aimed to explore whether parent–adolescent conflict and violent tendencies mediate the association between childhood maltreatment and adolescent risky behavior. It also explored the mediating role of parent–adolescent conflict between childhood maltreatment and violent tendencies in adolescents. Firstly, findings indicated that childhood maltreatment significantly and positively predicted adolescent risky behavior. Consistent with these findings, previous research has found that experiences of maltreatment during childhood were associated with increased engagement in risky behavior such as substance abuse, suicidal tendencies, and antisocial behavior [8,10,11,12,13,56]. Theoretically (e.g., general strain theory), the literature suggests that childhood maltreatment generates negative emotions, which young people may attempt to cope with through deviant behavior [25,26]. Additionally, empirical evidence supports that the emotional and psychological strain resulting from maltreatment may disrupt emotional regulation skills [3,34] and coping mechanisms [3,25,26], thereby increasing the likelihood of maladaptive behavior as a form of escape or self-harm [8,10,57], which is a risk factor for adolescent risky behavior. For instance, Lansford et al. [10] found that physical abuse in childhood was associated with aggression and criminal behavior in adolescence, illustrating how abuse creates psychological tension that leads to risky behavior. In conclusion, these findings suggest that childhood maltreatment may be a contributing factor to the development of risky behavior in adolescence. It also emphasizes the importance of intervention services to prevent risky behaviors, as well as the need for education and support for children exposed to childhood maltreatment.

Next, it was found that parent–adolescent conflict mediated the relationship between childhood maltreatment and violent tendencies and adolescent risky behavior. Consistent with these findings, GST suggests that the additional strain from family conflict serves as a significant source of stress [26], amplifying the effects of childhood maltreatment and increasing the propensity for violence and risky behavior. Similarly, previous research has shown that conflicts between adolescents and their parents, which are common during the search for independence and identity formation, can exacerbate the emotional strain experienced by adolescents [27,29]. Moreover, adolescents’ search for independence under the influence of maltreatment experiences can increase tensions of these conflicts [58,59] and create persistent conflicts, which is a risk factor for violent tendencies and risky behavior in adolescence [4,8,22,28]. This increased conflict may undermine trust and communication within the family and also decrease family support, further exacerbating negative emotional states and leading to increased violent tendencies and engagement in risky behavior to cope [5,8,22,34]. These findings underline the influence of family dynamics in the complex interaction between the effects of childhood maltreatment and adolescent risky behavior. This result underscores the need for trauma-informed practices that focus on strengthening family communication and providing emotional support to adolescents who have experienced maltreatment. Educators, counselors, and mental health professionals could benefit from targeted training to identify and address the unique needs of these adolescents, facilitating healthier coping strategies and reducing the likelihood of risky behaviors.

Finally, it was found that violent tendencies mediated the relationship between childhood maltreatment and adolescent risky behavior. According to previous research, adolescents who have experienced maltreatment are more likely to develop aggressive and violent tendencies as a result of the strain and emotional dysregulation caused by their adverse experiences [34,60]. This propensity for violence can be understood through GST, which posits that those high levels of strain lead to negative emotions and, consequently, aggressive behavior [61,62]. Therefore the chronic stress and trauma from maltreatment disrupt normal emotional regulation and coping mechanisms, resulting in a higher likelihood of violent behavior [42] as a means of expressing frustration and anger, which can manifest in various risky behavior, such as physical fights, substance abuse, and criminal activities [8,47]. Additionally, aggressive behavior can become learned habitual responses to stress and conflict [40], potentially spreading to other forms of risky behavior. Moreover, maltreated adolescents who have violent tendencies may be more likely to engage in risky behavior if they are in environments where such behavior is normalized [12]. The findings suggest that addressing the underlying strain and developing healthier coping mechanisms are essential for mitigating the short- and long-term impact of childhood maltreatment on adolescent behavior. Interventions that identify and address violent tendencies early can help youth develop healthier behaviors. Schools and community support systems can play a critical role in implementing these interventions by providing a supportive environment to reduce the risk of aggressive and risky behaviors among maltreated youth.

## 8. Limitations and Conclusions

This study has several limitations that need to be acknowledged. First, the cross-sectional design limits the ability to infer causality between childhood maltreatment, parent–adolescent conflict, violent tendencies, and adolescent risky behavior. Longitudinal studies are needed to establish temporal relationships and causality. The second limitation of this study is related to the qualities measured by the scale used to measure childhood maltreatment. Although the measure covers dimensions of maltreatment such as physical, emotional, and sexual abuse and neglect, it does not measure other dimensions that are currently accepted as child maltreatment, such as exposure to domestic violence. Future studies should consider including measures that capture a broader range of maltreatment experiences. The third limitation of this study is that the number of girls in the sample group is higher than that of boys. This may affect the reliability of gender comparisons. Therefore, the model was tested without controlling for gender. Future studies can test the model with gender as a control variable. Another limitation of the data was that they were collected using self-report measures, which may be subject to social desirability bias and recall inaccuracies. Participants may under-report or over-report their experiences and behavior, which could have an impact on the validity of the findings. Future studies should consider incorporating multiple data sources, such as observational methods or reports from parents and teachers, to triangulate findings and reduce bias. In addition, other potential confounding variables that may influence the relationships observed in this study, such as peer influence, coping strategies, and emotion regulation skills, were not considered in this study. Consideration of these variables in future research could provide a more comprehensive understanding of the factors influencing adolescent risky behavior.

In conclusion, this study contributes to the growing body of literature on the impact of childhood maltreatment on adolescent risky behavior by highlighting the mediating role of parent–adolescent conflict and violent tendencies. The findings underscore the importance of addressing the emotional and psychological strain caused by childhood maltreatment to mitigate its impact on adolescents’ propensity for risky behavior. These results suggest that interventions aimed at reducing strain and improving emotional regulation and coping mechanisms could be effective in preventing risky behavior in adolescents. This study emphasizes the need for comprehensive interventions that involve not only the affected adolescents but also their families to address underlying family dynamics and reduce conflict. By promoting healthier family relationships and emotional regulation skills, it may be possible to break the cycle of maltreatment, conflict, and risky behavior, leading to better developmental outcomes for adolescents. Future research should continue to explore these relationships and develop targeted interventions to support at-risk youth and their families.

## Figures and Tables

**Table 1:** Descriptive statistics and correlations for the study variables.

	Skewness	Kurtosis	Mean (Female)	SD (Female)	Mean (Male)	SD (Male)
Risky behavior	1.27	2.51	70.02	21.03	71.40	23.75
Parent–adolescent conflict	1.30	1.70	18.50	6.29	18.74	6.07
Violent tendency	0.691	0.701	32.22	6.77	35.41	8.53
Childhood maltreatment	0.869	−0.415	67.06	22.35	74.30	22.22
			1.	2.	3.	4.
1. Risky behavior			-	0.54 **	0.51 **	0.20 **
2. Parent–adolescent conflict				-	0.49 **	0.27 **
3. Violent tendency					-	0.22 **
4. Childhood maltreatment						-

** Correlation is significant at the 0.001 level (2-tailed).

**Table 2:** Unstandardized coefficients for the mediation model.

Antecedent	Consequent					
	Coeff.	*SE*	*t*	*p*	BootLLCI	BootULCI
	M_1_ (Parent–Adolescent Conflict)					
*X* (Childhood Maltreatment)	0.24	0.05	4.90	0.000	0.14	0.33
Constant	−0.02	0.05	−0.50	0.619	−0.12	0.07
	R^2^ = 0.07					
	F = 24.02; *p* < 0.001					
	M_2_ (Violent Tendency)					
*X* (Childhood Maltreatment)	0.12	0.05	2.27	0.023	0.01	0.22
M_1_ (Parent–Adolescent Conflict)	0.39	0.06	6.61	0.000	0.28	0.51
Constant	0.00	0.05	001	0.990	−0.10	0.10
	R^2^ = 0.17					
	F = 30.85; *p* < *0*.001					
	Y (Adolescent Risky Behavior)					
*X* (Childhood Maltreatment)	0.01	0.04	0.30	0.761	−0.07	0.10
M_1_ (Parent–Adolescent Conflict)	0.44	0.05	8.20	0.000	0.33	0.55
M_2_ (Violent Tendency)	0.34	0.05	7.08	0.000	0.25	0.44
Constant	0.00	0.04	0.05	0.956	−0.08	0.09
	R^2^ = 0.40					
	F = 67.42; *p* < 0.001					

Note: *SE* = standard error; Coeff = unstandardized coefficient; *X* = independent variable; M = mediator variables; Y = outcome or dependent variable.

**Table 3:** Unstandardized total, direct, and indirect effects for the mediation model.

Path	Effect	*SE*	BootLLCI	BootULCI
Total effect	0.19	0.05	0.08	0.29
Direct effect	0.01	0.04	−0.07	0.10
Total indirect effect	0.18	0.04	0.10	0.26
CM → PAC → ARB	0.10	0.02	0.05	0.16
CM → VT → ARB	0.04	0.03	0.00	0.07
CM → PAC → VT→ ARB	0.03	0.02	0.01	0.06

Note: CM, childhood maltreatment; PAC, parent–adolescent conflict; ARB, adolescent risky behavior; VT, violent tendency; number of bootstrap samples for percentile bootstrap confidence intervals: 5000.

## Data Availability

Data are available upon reasonable request.
